# Nanorobot-guided modulation of microglial phenotypes: a promising strategy to mitigate neuroinflammation in traumatic brain injury

**DOI:** 10.1097/MS9.0000000000003779

**Published:** 2025-08-29

**Authors:** Muhammad Daim Hassan, Muhammad Usman, Maliha Khalid, Muhammad Talha, Aminath Waafira

**Affiliations:** a Department of Medicine, King Edward Medical University, Lahore, Pakistan; b Department of Medicine, Jinnah Sindh Medical University, Karachi, Pakistan; c Department of Medicine, The Maldives National University, Malé, Maldives

**Keywords:** microglial modulation, nanorobots, neuroinflammation, traumatic brain injury

## Abstract

Traumatic brain injury (TBI) triggers neuroinflammation largely driven by microglial activation, where prolonged pro-inflammatory (M1) phenotypes exacerbate neuronal damage. Nanorobot-mediated delivery systems represent a promising frontier in targeting this inflammation by crossing the blood–brain barrier and precisely modulating microglial behavior. These nanorobots, engineered to identify diseased tissues and release anti-inflammatory agents, can effectively shift microglial states toward the reparative M2 phenotype. Recent advancements, such as DNA-based nanorobots and biomimetic microrobots, have demonstrated significant neuroprotective effects in preclinical models. Despite ongoing challenges around biosafety, biocompatibility, and scalability, this therapeutic paradigm holds substantial potential for transforming TBI treatment. Collaboration between developers and clinicians is vital for refining nanorobotic technologies and enabling their safe, effective clinical deployment in neuroinflammation control.

*To the Editor,*

Traumatic brain injury (TBI) occurs due to a significant impact to the head and can lead to brain hemorrhage, brain dysfunction, or even death. It causes damage to neurons, supporting cells, and blood vessels. TBI may manifest as neuroinflammation, marked by the activation of resident glial cells, the release of inflammatory mediators in the brain, and the infiltration of peripheral leukocytes. While microglia initially play a central role in clearing debris and promoting repair, their sustained pro-inflammatory activation can worsen neuronal damage. Current therapeutic approaches often lack efficacy due to their limited ability to cross the blood–brain barrier (BBB) and achieve precise modulation of microglial phenotypes^[1]^.

Nanorobot-mediated modulation of microglia is a recent advancement in nanotechnology that provides a novel curative pathway for TBI-mediated neuroinflammation. A nanorobot is a small-scale structure that is able to carry out a preprogrammed task through mechanical actuation^[2]^. The surface of the nanorobot is directly coated with pharmaceutical agents by electrostatic interactions. Nanorobots are programmed to identify specific markers of diseased cells. Once they find their target, they release the medication directly into the affected cells. This not only maximizes the drug’s impact but also minimizes harm to healthy tissues. Microglia in the brain adopt either pro-inflammatory (M1) or anti-inflammatory (M2) phenotypes in response to injury. The prolonged M1 activation may aggravate neuronal damage post-TBI. Thus, the shifting of microglia cells toward the M2 phenotype is crucial for minimizing inflammation and maximizing neuronal repair^[3]^.

Nanorobots act as an accurate and precise vector for delivering modulatory agents directly to microglial targets across the BBB. In recent years, several advancements have been made in this avenue. For example, tetrahedral DNA nanorobots loaded with siCCR2 were introduced in 2021, which demonstrated effective polarization of microglia toward M2 activity in intracerebral hemorrhage models, a therapeutic technique likely applicable to TBIs^[4]^. Similarly, oxidative stress and neuroinflammation were suppressed by balloon-coated nanomotors through modulation of microglial polarization. Biomimetic microrobots coated with cell membranes can avoid immune detection and precisely deliver anti-inflammatory molecules to injured sites^[5]^.

Despite the progress of nanorobots in the last decade, one of the significant challenges of this field lies in applying these tools to widespread clinical use. Nanorobots are required to perform essential functions, including targeting, energy supply, driving, and biocompatibility. However, the key goal remains to achieve a specific environment and functional application for nanorobots. Also, ensuring the biosafety of these nanorobots is an important target for the developers. Materials used in contemporary nanorobots might also potentially have hazardous properties that require further investigation. Allergenic reactions have been observed in people working with nano-sized nickel powder^[6,7]^.

In conclusion, the integration of neuroscience, nanorobotics, and immunomodulation promises a more effective and efficient change in TBI therapeutics. This innovative approach holds the potential to not only control acute neuroinflammation but also to prevent chronic neurodegeneration. To fully unlock its true potential, the developers and physicians should collaborate in the research trials and refinement of this therapeutic innovation, thus fostering a new era of nanorobotic technology-based TBI and neuroinflammation control. Our work is in line with the TITAN Guidelines on the need for transparency in AI use in healthcare^[8]^.

## Data Availability

No data were generated for this manuscript.

## Research registration unique identifying number (UIN)

Name of the registry: N/A. Unique Identifying number or registration ID: N/A. Hyperlink to your specific registration (must be publicly accessible and will be checked): N/A.
